# From microseconds to seconds and minutes—time computation in insect hearing

**DOI:** 10.3389/fphys.2014.00138

**Published:** 2014-04-11

**Authors:** Manfred Hartbauer, Heiner Römer

**Affiliations:** Institute of Zoology, Karl-Franzens University GrazGraz, Austria

**Keywords:** interaural time difference, directional hearing, signal timing, chorus synchrony, mate choice, precedence effect, time window

## Abstract

The computation of time in the auditory system of insects is of relevance at rather different time scales, covering a large range from microseconds to several minutes. At the one end of this range, only a few microseconds of interaural time differences are available for directional hearing, due to the small distance between the ears, usually considered too small to be processed reliably by simple nervous systems. Synapses of interneurons in the afferent auditory pathway are, however, very sensitive to a time difference of only 1–2 ms provided by the latency shift of afferent activity with changing sound direction. At a much larger time scale of several tens of milliseconds to seconds, time processing is important in the context species recognition, but also for those insects where males produce acoustic signals within choruses, and the temporal relationship between song elements strongly deviates from a random distribution. In these situations, some species exhibit a more or less strict phase relationship of song elements, based on phase response properties of their song oscillator. Here we review evidence on how this may influence mate choice decisions. In the same dimension of some tens of milliseconds we find species of katydids with a duetting communication scheme, where one sex only performs phonotaxis to the other sex if the acoustic response falls within a very short time window after its own call. Such time windows show some features unique to insects, and although its neuronal implementation is unknown so far, the similarity with time processing for target range detection in bat echolocation will be discussed. Finally, the time scale being processed must be extended into the range of many minutes, since some acoustic insects produce singing bouts lasting quite long, and female preferences may be based on total signaling time.

## Introduction

Some insect taxa use acoustic signals for intraspecific communication, similar to anurans, birds and mammals (Bradbury and Vehrencamp, 2011). The temporal pattern of these signals plays a key role, as it is the main carrier of information for species identification and for discrimination between mates (Gerhardt and Huber, 2002; Greenfield, 2002). Time computation in processing these sounds by the auditory system is critical in several aspects:

The songs exhibit a stereotyped temporal pattern, where either single pulses are repeated at a rate of about 10–200 Hz, or the pulses are grouped into chirps, repeated at varying rates. This is typical for all insects studied: crickets (Alexander, 1962; Otte, 1992), katydids (Schul, 1998), grasshoppers (von Helversen, 1972) and fruit flies (Hoy et al., 1988). Since the behavior of receivers in response to these temporal patterns is also rather stereotyped and expressed either as a selective phonotaxis or phonoresponse, the temporal selectivity has been extensively studied with song models, using different behavioral paradigms such as trackballs, Kramer treadmills or in arena trials. In addition, behavioral data have been complemented by classical neuroethological studies to identify the neuronal basis for the temporal specifity observed in behavior, and even robotics added to our understanding of temporal processing of insect song patterns (Webb, 2002). This aspect of time computation deserves a separate review and is not covered here, but the interested reader can find reviews in Gerhardt and Huber (2002; Hedwig, 2006) and the most recent results on crickets (Clemens and Hennig, 2013) and grasshoppers (Clemens and Ronacher, 2013) demonstrate that time computation of song patterns happens at two time scales: a short one associated with the extraction of stimulus features such as pulse duration and pulse interval, and a long one for the integration of these features over the whole time of the song. Kostarakos and Hedwig (2012) provide new insights into the neuronal network in the cricket brain responsible for the species-specifity of the field cricket calling song.

Like all other animals equipped with two ears (for exceptions see Yager and Hoy, 1987; van Staaden and Römer, 1998), insects could make use of binaural cues for sound localization. However, the small size of insects and thus the small interaural distance between the ears results in only minute interaural time differences (ITDs). In crickets, for example, the range is only about 5–23 μs (calculated from distances between ears in the smallest and largest cricket species at an angle of sound incidence of 45°), so that ITDs appear not to be available for sound localization. We review here how insects may overcome such limitations, by processing the difference in the time of arrival of binaural afferent activity, rather than the physical time delays between the ears. Nevertheless, the case of an insect with hyperacute directional hearing, the parasitoid fly *Ormia ochracea*, demonstrates that time differences in the sub-microsecond range can be used, given that some adaptations in the physiology of receptors are met (for review see Robert, 2005).

Whereas the range of time differences relevant for directional hearing in insects is from below a microsecond to a few milliseconds, there are time differences in the order to tens of milliseconds to more than 100ms which are important when it comes to the interactions of signals between singing males. Insects often sing in choruses, where individual song elements are not produced randomly relative to competing signalers, but follow specific temporal patterns (Greenfield, 1994b, 1997). As an example we describe here the case of imperfect acoustic synchrony in a katydid, and how the enhanced directionality of interneurons in the auditory pathway due to lateral inhibition contributes to the asymmetrical representation of differently timed signals in receivers. This in turn may affect the choice of females toward signals of males leading in time.

A very special case of time computation occurs in a group of katydid, where acoustic communication involves a duet between the sexes, and where species identification is not the result of processing a species-specific temporal pattern of song. Rather, the specifity is achieved by a rather narrow time window for the female reply; any response outside this window does not elicit a phonotactic response of the male (review Bailey, 2003). Although the neuronal implementation of such time windows is currently not known, we discuss this special case of time computation as it provides some unique properties unknown from any vertebrate hearing system.

## Physical and physiological interaural time differences as cues for directional hearing

As for all other bilaterally symmetrical animals, almost all insects for which the sense of hearing has been documented have one pair of ears, and could thus use binaural cues to determine the location of sounds in the azimuth [for exceptions in the praying mantis with only one ear see Yager and Hoy, 1987, and bladder grasshoppers (Pneumoridae) with six pairs of ears see van Staaden and Römer, 1998]. Theoretically, therefore, the auditory system of insects could employ the interaural intensity differences (IIDs) that result from the inherent directionality of the ear, as well as ITDs due to sound reaching one ear earlier than the other when the angle of sound incidence is off the symmetry axis of the body. The amount of ITDs thus created is small even in mammals and depends on head size (humans: 600 μs; gerbils about 150 μs). However, in insects the interaural disparity can be extremely small: in many of the short-horned Acridid grasshoppers, the tympana of the ears in the first abdominal segment are no more than 1–2 mm apart, hence the available ITDs amount to only 3–5 μs. As an extreme case, the best possible ITD in the parasitoid fly *Ormia ocracea* has been measured with 1.45 μs (Robert et al., 1996; Mason et al., 2001; see below). Similarly, the distance between the ears in the forelegs of small crickets and katydids is minute as well, so that the use of ITDs as a cue for sound localization is so strongly constrained by body size and ear separation, that it is generally accepted that such small ITDs cannot be used for neuronal processing of sound direction.

The pressure gradient receiver of crickets is the best studied case where phase relationships play an important role for directional hearing (Michelsen et al., 1994; Michelsen, 1998). Their hearing organs are located in the front legs, and sound can act on the outer surface of the tympanum, but also on the inner surface through an acoustic trachea connecting the inner surface of the tympanum with an ipsilateral spiracle at the body surface. Furthermore, there is an important transverse tracheal connection between both sides, so that, theoretically, the cricket ear has four acoustic inputs (Michelsen et al., 1994). The phase delay of the sound reaching the eardrum from the ipsilateral spiracular opening and acoustic trachea increases about 18° per kHz, but the phase of sound transmitted from the contralateral spiracle through the transverse trachea change with more than 100° per kHz at frequencies between 3 and 10 kHz. A central double membrane located in the midline of the transverse trachea appears to be crucial for establishing such large phase delays (Michelsen and Löhe, 1995).

**Figure 1:**
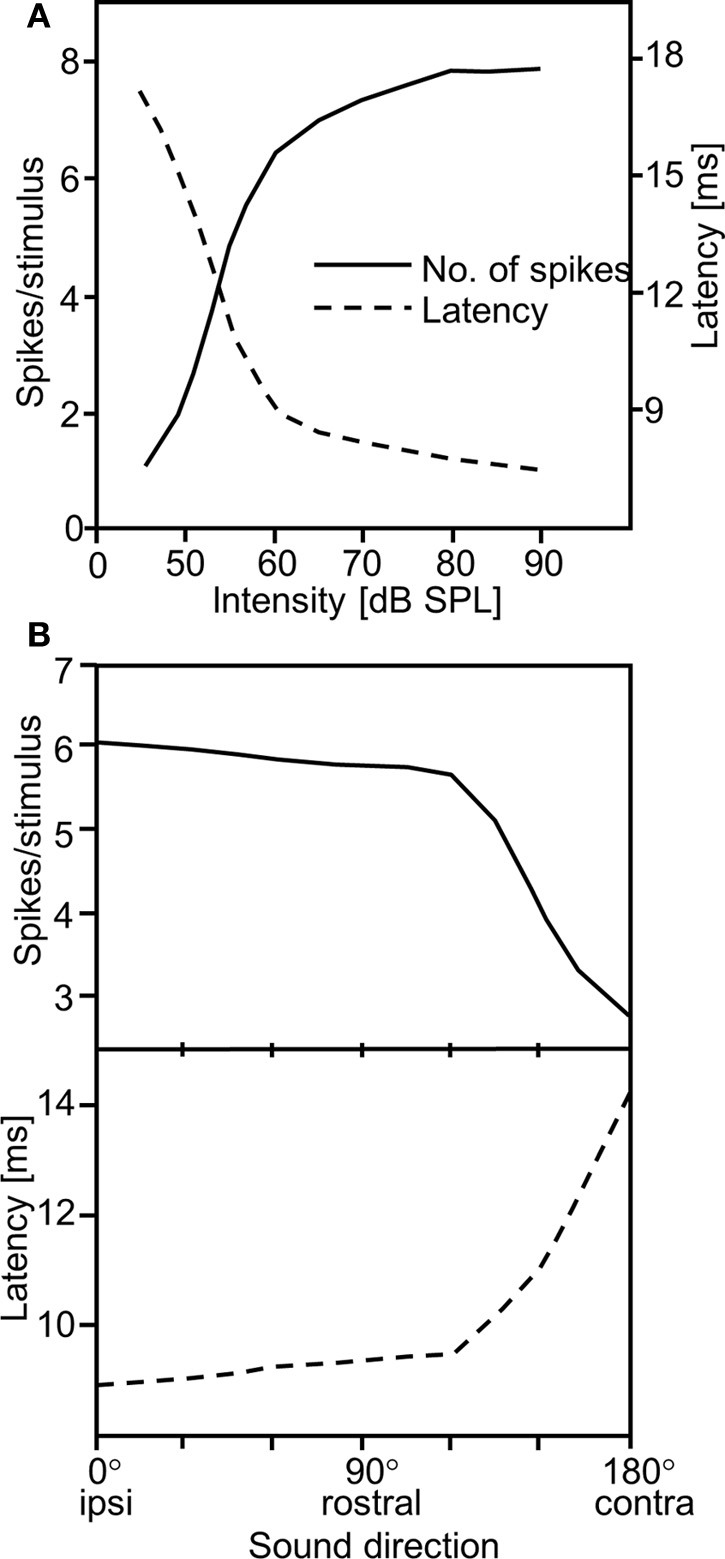
**(A)** Inverse relationship of spike count and response latency in locust auditory receptors. **(B)** As a result of the high correlation between both, two directional cues are available for the central nervous system when the sound source is moved from ipsi- to contralateral: spike count and latency differences (modified from Mörchen et al., 1978).

Despite physical limitations arising from a small distance between ears, insects can localize their mates (or hosts in the case of parasitoids) quite well (Gerhardt and Huber, 2002, for review). This could be due to the fact that IIDs as a result of the ears' directionality are sufficient for this task. However, Mörchen et al. (1978) suggested a different solution, by examining the well-known effect that the latency of sensory excitation is dependent on stimulus intensity (Figure 1A; for a similar finding in crickets see Imaizumi and Pollack, 2001). If the directionality of the ear creates IIDs, due to the high negative correlation between response strength and latency, this would result in a direction-dependent latency shift in auditory nerve fibers (Figure 1B). Thus, the difference in the time of arrival of action potential activity on both sides of the nervous system is about 5–6 ms for ipsi- vs. contralateral sound, which is almost 1000 times the value of the physical time delay between the ears. Response strength and response latency can therefore be regarded as equivalent directional cues for sound direction (Mörchen et al., 1978).

**Figure 2:**
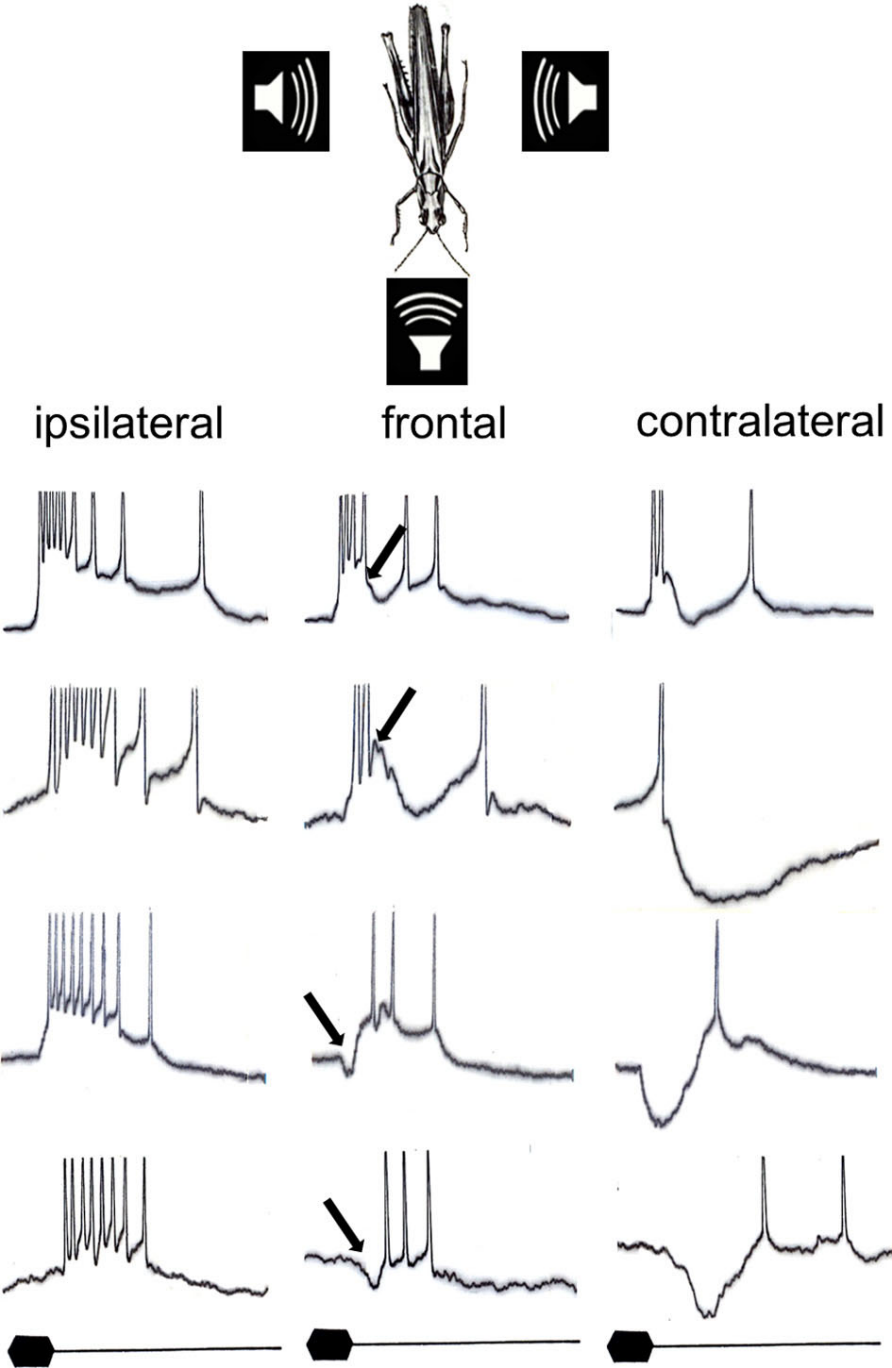
**Representative intracellular responses of four different interneurons in the afferent auditory pathway of the locust for sound stimuli presented from their respective ipsilateral, frontal, and contralateral side**. Note that all neurons have in common excitatory input from ipsilateral and inhibitory input from contralateral, but that both the timing and the strength of inhibition result in a magnification of either latency differences or spike count differences with changing sound direction (modified from Römer et al., 1981). The arrows indicate the onset of inhibition, which is delayed relative to the excitation in neurons #1 and #2, but is elicited earlier than the excitation in neuron #3 and #4. Stimulus duration: 20 ms.

How are these cues represented in the network of local and ascending interneurons? Mörchen (1980) examined the directional patterns for these cues and found again an inverse correlation in most directionally sensitive interneurons, but with a much higher degree of latency shift in the order of 20 ms, when the sound source was moved from ipsi- to contralateral. In other interneurons, either spike count or response latency was affected by sound direction. Thus, in interneurons providing the afferent information about the direction of sound sources the cues of the auditory receptor fibers may be enhanced compared to the periphery, but differently expressed as a result of different types of binaural synaptic interactions. Intracellular studies revealed the underlying mechanism for the different directional patterns of interneurons in the locust (Römer et al., 1981; Figure 2). Although they have in common the excitatory input from ipsilateral and inhibitory input from contralateral, the timing of inhibition relative to excitation is important: early inhibitory potentials relative to the excitation delay the latency of action potentials strongly, whereas a delayed contralateral inhibition only influences the response strength, but not the response latency. The amount of inhibition appears to be responsible for the variation in the directionality of the spike count (compare for example the first two interneurons in Figure 2).

**Figure 3:**
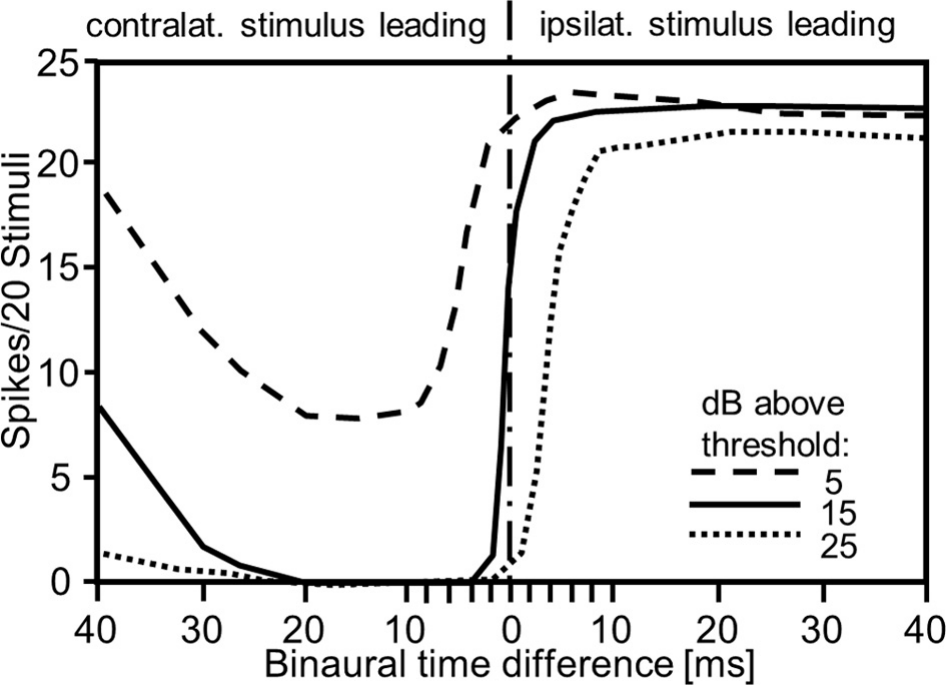
**Effect of interaural time and intensity differences on the response of an auditory interneuron in the locust**. Note that the steepest slopes of the curves are produced by small interaural time differences in the order of 1–2 ms, such as those provided by auditory receptors (compare with Figure 1B), and that the slopes can be traded by changing interaural intensity differences (modified from Rheinlaender and Mörchen, 1979).

In a series of elegant experiments using dichotic stimulation of tympana in the locust ear with piecoelectric transducers Rheinlaender and Mörchen (1979) examined the influence and the sensitivity of the dual mode of directional coding for central interneurons. They demonstrated a time-intensity trading phenomenon similar to the one reported for vertebrates, although the time cue is in the order of milliseconds and not microseconds. Remarkably, shifting the contralateral stimulus only 2–4 ms ahead of the ipsilateral one changed the activity of an interneuron from maximal excitation into total inhibition (Figure 3). This effect could be traded by intensity of either stimulus. In another experimental approach, Römer and Rheinlaender (1983) used electrical stimulation of the tympanal nerve of the locust to manipulate spike rate on one side without affecting the response latency. Their result indicated that spike rate alone could also be sufficient for directional coding. In this way, all these experiments confirm that the dual mode of directional coding provided by auditory receptor fibers is indeed used at the first site of synaptic processing, and although microseconds do not matter because the physical time delays between the ears are too small to be of relevance, the onset of binaural arrival of receptor activity at these synapses (the physiological time delay) is quite important for directional coding.

**Figure 4:**
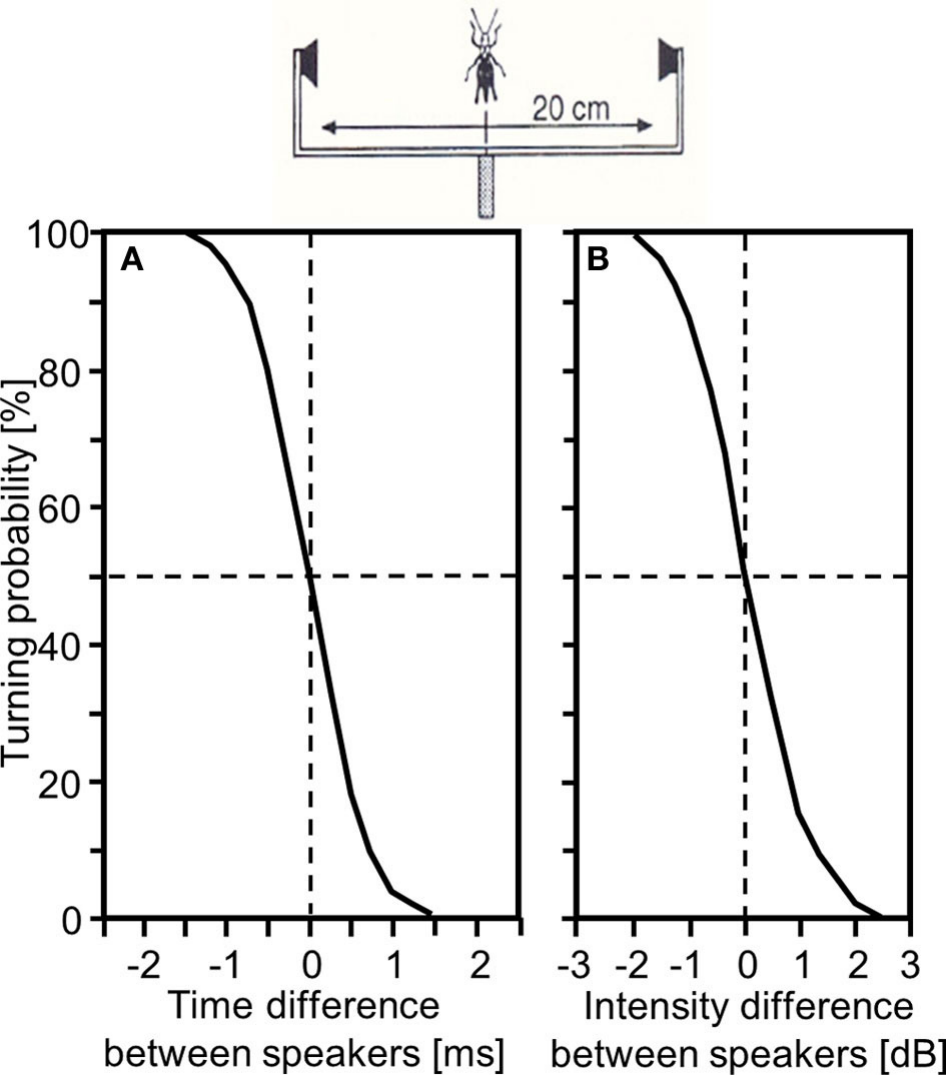
**Behavioral demonstration that small interaural time differences (A) or interaural intensity differences (B) in the order of only 1 ms and 1 dB influence the turning tendency of male *Chorthippus biguttulus* in a quasi-dichotic ear stimulation experiment (modified from von Helversen and Rheinlaender, 1988)**.

In a quasi-dichotic stimulus situation via bilaterally arranged miniature speakers the behavioral resolution of a grasshopper male for ITDs was in the order of 1ms: when both stimuli were presented at equal loudness, but one speaker was leading the other by only 0.5 ms this resulted in significant turns toward the leading side (Figure 4A). In a similar experiment, when both stimuli were presented at the same time, a difference in intensity of 0.5 dB elicited significant turns to the louder side; IIDs of more than 1.5 dB elicited error-free turns (von Helversen and Rheinlaender, 1988; von Helversen, 1997; Figure 4B). Thus, there is also convincing behavioral evidence that both ITDs and IIDs, are used as cues for directional coding. Since the latter two experiments indicate that each afferent cue for sound direction alone (namely binaural differences in spike number and in the latency of spikes) may be sufficient for correct lateralization of a stimulus, we may ask whether at all, or under which circumstances, both cues are necessary. Previous experiments by Givois and Pollack (2000) have shown that these two cues are affected differently by intense prior stimulation. Thus, Pollack (2003) used this phenomenon as a tool to uncouple interaural differences in response strength and latency, and determined the consequences for sound localization through phonotactic responses of crickets. His results indicated that the dominant sensory cue for sound location is interaural difference in response strength.

Hearing conditions outdoors with respect to directional cues can be poor and unpredictable for receivers approaching a sound source (see Kostarakos and Römer, 2010 for field crickets), so that redundancy in using both cues appears to be a solution. However, we would argue that this does not improve the quality of directional hearing, since latency and the number of spikes are negatively correlated (Figure 1A), and if for whatever reason there is “wrong” directional information at a given receiver position, this will be translated into “wrong” spike count and latency information.

As mentioned above, the ear of the fly represents an extreme case of small ITDs: the interaural distance of 520 μm would create no more than 1.45 μs acoustical ITD. However, these ears are mechanically coupled pressure receivers. The coupling of the tympana by a flexible cuticular lever has the effect that the two tympanal membranes move out of phase at frequencies relevant for the fly (the CF of the cricket song; Miles et al., 1995; Robert et al., 1996). The mechanical ITD of 50 μs is much larger than the acoustical ITD of 1.45 μs. However, when these flies have been tested on a trackball system they showed a remarkable, hyperacute directionality: they oriented reliably toward the sound source even when the deviation from the midline was only 1°–2° (Mason et al., 2001). With such small stimulus angles, the flies have to deal with acoustical ITDs as small as 50 ns, and 2 μs for the mechanical ITD. Physiological recordings of activity of primary afferents of the fly have shown that the remarkable temporal resolution is based on a number of properties, such as phasic responses with very high temporal acuity, no spontaneous activity, and again (see Figure 1A) an inverse relationship of spike latency and stimulus intensity (Mason et al., 2001; Oshinsky and Hoy, 2002).

## Time windows—an unusual problem of time computation in acoustic communication

In the vast majority of grasshoppers, crickets, katydids and cicadas acoustic communication is characterized by a male producing a calling song and a receptive but mute female performing phonotaxis toward the stationary male. As pointed out by Zimmermann et al. (1989) the males in these cases are “speculative signalers” since they do not know the effectiveness of their signaling until the very last moment of arrival of a female. Some short-horned grasshoppers and katydids have evolved a different communication system, where pair formation is achieved by duetting: the male song elicits an acoustic reply from the female. Either the male (in most species) or the female then respond phonotactically (von Helversen, 1972; Hartley et al., 1974; Heller and von Helversen, 1986; Zhantiev and Korsunovskaya, 1986; review by Bailey, 2003). In the context of time computation the duetting in Phaneropterine katydids is of special interest because of three specific attributes, which place a high demand on both the temporal sensitivity and the precision in the acoustic behavior of both sexes: (1) The song duration of both sexes is often unusually short in the order of a few milliseconds; in *Leptophyes punctatissima* the female reply is a short click less than 0.5ms in duration. (2) The female response to the male call in this species occurs after a very short delay time of about 28 ms (Robinson et al., 1986), remarkably constant for each individual. In *Andreiniimom nuptialis* the delay is even shorter, around 18 ms; it represents one of the fastest acoustico-motor responses known among insects (Heller and von Helversen, 1986). (3) The female reply has to occur within a time window from about 25 to 45 ms after onset of the male song in order to elicit phonotaxis in the male (Robinson et al., 1986).

All three temporal attributes are interrelated. Since the female reply is an extremely short click, species-specific identification is a problem since such a short click does not provide the species-specific amplitude modulations usually necessary to distinguish between songs of species. However, the delay time of the female is a very precise species-specific characteristic, varying between species from less than 20 up to 450 ms, and could be used by the male as a temporal feature for species recognition (Heller and von Helversen, 1986). When the delay time was varied experimentally, males revealed narrow and species-specific time windows for the female reply to elicit phonotaxis (Heller and von Helversen, 1986; Robinson et al., 1986; Dobler et al., 1994). It has been speculated by these authors that the combination of extremely brief signals, a narrow time window of the male and the corresponding delay time of the female may be advantageous under noisy field conditions, since listening for, and only accepting, a signal in a narrow time window may reduce many false alarms. This has, however, never been tested under realistic outdoor conditions and awaits further experimental proof.

Another outstanding feature of the communication scheme in Phaneropterine katydids makes them unique compared to all hearing animals: due to the narrow male time window the travelling time of sound through air becomes a significant fraction of the female delay time as being perceived by the male, and may therefore limit the maximum communication distance between the sexes. Zimmermann et al. (1989) examined the limitations given by either travelling time or attenuation of the call experimentally. For each meter increase in communication distance the overall time delay increases by about 6 ms (transmission of male signal to female, and female reply back to male). For a female with a delay of 28 ms a maximum distance of 4.5 m can be calculated, corresponding to the outer edge of the male's time window. By manipulating delay times and intensities independently, the authors demonstrated that in about 1/3rd of duets only intensity was the limiting factor, in the remaining ones it was time and intensity. Also remarkable was the sharp drop-off in phonotaxis with an increase of the female overall response delay by 3 ms beyond a critical value, or a decrease in sound intensity by only 1.5–2.0 dB below the behavioral threshold (Zimmermann et al., 1989).

Although such time windows have been known for quite a long time, at present the way in which this time information is implemented in the nervous system of an insect is completely unknown. The necessary neurophysiological experiments are not easy to perform because they would require a restrained singing male while searching intracellularly (most likely in his brain) for a mechanism that favors a response to a female reply only 25–45 ms after his call. An intriguing possibility might be that temporal selectivity is facilitated by the timing of excitation which coincides with postinhibitory rebound excitation, as modeled by Large and Crawford (2002). A local, non-spiking brain neuron was recently found in the brain of a field cricket with such properties, where the timing is critical for tuning of the temporal chirp pattern (Kostarakos and Hedwig, pers. communication). Interestingly, the selectivity of these time windows is also analogous to the coding of target distance in echolocating bats, where the delay between the emitted sound pulse (the male call in the case of the katydid) and the returning echo (equivalent to the female reply) conveys information about target distance (Suga, 1990). In the medial geniculate body and the auditory cortex of the bat neurons have been found that respond only when pulse and echo are combined with a particular delay time (“combination-sensitive” neurons or FM-FM-cells; Suga et al., 1978; O'Neill and Suga, 1982). Similarly, one would expect cells in the brain of Phaneropterine male katydids that respond only when the delay between the own call and the female reply has the species-specific delay (see Carr, 1993 for a discussion about the underlying delay lines in bats).

## The importance of signal timing for mate choice

We have shown above that signal timing is the only cue for mate recognition in duetting species because males expect a female reply within a certain species-specific time window. However, signal timing is also an important signal feature in other acoustically communicating species where males congregate in groups forming so-called acoustic leks or “spree” and females select among competing males (Walker, 1983; Kirkpatrick and Ryan, 1991; Höglund and Alatalo, 1995). Males calling in aggregations offer females the possibility to compare the calling songs of potential mates simultaneously, as opposed to sequential mate choice where receivers are challenged in memorizing the calls of different individuals (Kokko, 1997). In a chorus, signal timing among males often deviates strongly from random so that various temporal structures of collective broadcast emerge. In some species females select males on the basis of relative signal timing rather than other signal features (Greenfield, 1994a; Gerhardt and Huber, 2002). Such mating systems are especially interesting for evolutionary biologists since females seem to gain no obvious fitness benefits by choosing males on this basis (Alexander, 1975; Greenfield, 1994a).

Some of these chorusing insect species collectively broadcast acoustic (or visual in the case of fireflies) mating displays in almost perfect synchrony, resulting in fascinating group displays (Fireflies: Buck and Buck, 1968; Otte and Smiley, 1977; Buck et al., 1981; Orthoptera: e.g., Walker, 1969; Sismondo, 1990; Greenfield, 1994b; Nityananda and Balakrishnan, 2007). Synchronized signaling is not restricted to the acoustic and visual world of insects, but can be found in the vibratory and visual communication systems of wolf spiders (Kotiaho et al., 2004) and fiddler crabs (Blackwell et al., 1998). By contrast, a low degree of temporal signal overlap is usually found in most frog choruses, where males avoid acoustic overlap with neighbors by either timing their signals in a “forbidden interval” at the end of competing signals (Greenfield, 1994a; Höbel and Gerhardt, 2007), or signal in “windows” of relatively low level of noise generated by conspecifics (Zelick and Narins, 1985; Schwartz, 1987; Brush and Narins, 1989). A characteristic of synchronizing species is their highly regular, periodic signal production. Signaling is controlled through a central pattern generator that leads to a very high precision of signal timing, if individuals in a group slowly adapt their signal period to the rhythm of neighbors with similar intrinsic “free-running” signal periods. In addition, some chorusing species are able to uphold a certain phase relationship between their signal rhythms by responding with a sudden phase shift to the signal of a neighbor. As a result signals are either broadcast in collective synchrony or in alternation with a phase shift of about 180°. In the katydid *Mecopoda elongata*, for example, solo singing males produce chirps at periods of about 2 s, with a coefficient of variation of signal period of only 2–3% (Hartbauer et al., 2005, 2012). In acoustic interactions, males establish chorus synchrony with a high degree of signal overlap, whereas in transient song episodes chirps are produced in alternation (Hartbauer et al., 2005).

Some attention has been paid in the past to the question of whether such synchronous displays result from cooperation or competition (review in Greenfield, 1994a). Males gain mutual benefits by attaining or conserving signal efficacy through cooperation if one or more of the following group benefits arise: (1) Synchrony preserves a species-specific temporal pattern (Walker, 1969; Greenfield and Schul, 2008). (2) Alternation ensures that females are able to detect critical signal features and localize individual signalers. (3) Synchrony maximizes the peak signal amplitude of group displays, (the “beacon effect”), thus increasing the conspicuousness of a group of synchronous males compared to lone singers. In turn a group of males may attract a higher number of females compared to solo singers. However, selective forces ultimately leading to signal timing that deviates from random can be manifold and act on individuals as targets of selection besides possible group benefits. For example, preferences of natural predators and conspecific females are known to exert a driving force for signal timing in a chorus, in particular if acoustically-orienting predators or parasitoids have difficulties in localizing any one signaler in a synchronous chorus owing to cognitive limitations (Otte, 1977; Tuttle and Ryan, 1982) and males profit from a reduced per-capita rate of predation (Lack, 1968; Wiley, 1991; Alem et al., 2011; Brunel-Pons et al., 2011).

In male aggregations of some anuran and katydid species sexual selection favored the evolution of oscillator properties enabling either synchronous or alternating signal displays. Such signal timing-adjustments emerged as the outcome of inter-male rivalry for mates and is driven by inter-sexual selection (e.g., Alexander, 1975; Greenfield, 1994a,b, 1997; Snedden and Greenfield, 1998; Gerhardt and Huber, 2002; Höbel and Gerhardt, 2007; Copeland and Moiseff, 2010). In species where females prefer non-overlapping calls, males avoid partial or complete signal overlap as well as a “forbidden interval” at the end of a competitor's signal (Greenfield, 1994a; Tauber et al., 2001; Höbel and Gerhardt, 2007). For example, many anuran species evolved this signaling strategy and form choruses in which the signals of neighbors frequently interchange (Zelick and Narins, 1985; Schwartz, 1993; Grafe, 1996; Gerhardt and Huber, 2002). In contrast, calling in synchrony in a male assemblage is usually of limited precision so that some males tend to time their signals slightly in advance to others following these signals. In some synchronizing species females prefer the leader of two otherwise identical signals, which forces males to compete for signals timed as leader in acoustic interactions. In these cases chorus synchrony is likely established as the evolutionary outcome of inter-male competition for females (Greenfield and Roizen, 1993; Greenfield, 1997; Snedden and Greenfield, 1998; Fertschai et al., 2007).

Examples for a preference of signals that are timed in advance to others (leader signals) can be found in many Orthoptera: [*Neoconocephalus spiza* (Greenfield and Roizen, 1993; Snedden and Greenfield, 1998), *Amblycorypha parvipennis* (Galliart and Shaw, 1991), *Ephippiger ephippiger* (Greenfield et al., 1997), *Ligurotettix planum* (Minckley and Greenfield, 1995) *Ligurotettix coquilletti* (Greenfield et al., 1997), crickets (Wyttenbach and Hoy, 1993)]. A preference for leader signals constitutes a precedence effect, which is defined as a receiver preference for the leading signal of two closely timed identical signals presented from different directions. The precedence effect is not restricted to insects but can also be found in humans, mammals, birds, frogs and invertebrates (humans: Zurek, 1987; Litovsky et al., 1999; Mammals, birds, frogs and fiddler crabs: Cranford, 1982; Klump and Gerhardt, 1992; Blackwell et al., 1998; Dent and Dooling, 2004; Marshall and Gerhardt, 2010).

**Figure 5:**
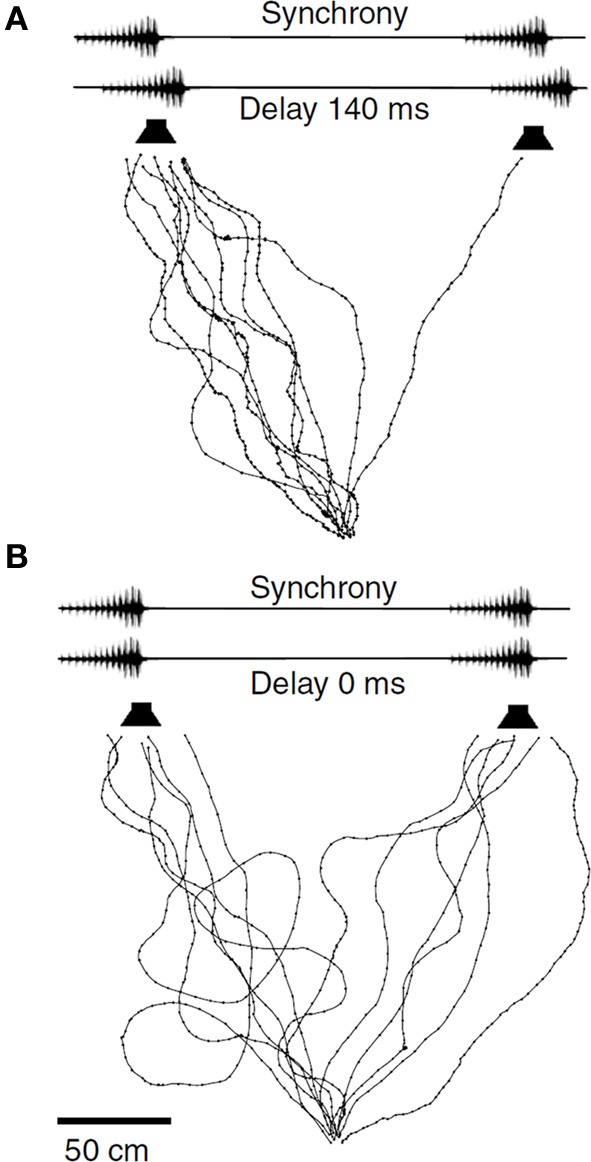
**Leader preference in *M. elongata***. Phonotactic walking paths of females given the choice between identical chirps either presented with a temporal advantage of 140 ms from the left **(A)** or in perfect synchrony **(B)** Modified from Fertschai et al. (2007).

In contrast to *N. spiza*, where the leader role frequently changes between acoustically interacting males, male *M. elongata* establish rather fixed temporal relationships of their signals for long periods of time (Hartbauer et al., 2005). In small choruses consisting of four male individuals often maintain either leader or follower roles for at least 50% of the duration of song bouts (Hartbauer et al., unpublished results). When females are given a choice between identical male chirps differing only in their temporal relationship females show a strong preference for leading chirps if the temporal advantage is between 70 and 140 ms (Figure 5; Fertschai et al., 2007; Hartbauer et al., unpublished). Similar to the time-intensity-trading reported in the context of directional hearing before, a neurophysiological approach in Mecopoda revealed that a stronger neuronal representation of the leader signal with a temporal advantage of 140 ms requires an increased loudness of follower signals by 8 dB to be equally well represented in the nervous system of receivers (Fertschai et al., 2007). Results of simulations with computer-based agents that implemented data based on these neuronal representations closely resembled decisions of real females for leader and follower signals under the various time-intensity trading conditions (Stradner, 2008). Such a trade-off between signal timing and amplitude is common among synchronizing insect, and some anuran species (Klump and Gerhardt, 1992; Greenfield, 1994a; Howard and Palmer, 1995; Grafe, 1996; Greenfield et al., 1997; Snedden and Greenfield, 1998; Nityananda et al., 2007; Höbel, 2010). The implication for the behavior under field conditions is that females have to be in close proximity to the follower in order to select this male instead of the leader of a chorus. This raises the question about song oscillator properties enabling *M. elongata* males to attain leadership.

## An oscillator property responsible for attaining leadership

The average chirp period of *M. elongata* males singing in acoustic isolation is 2 s at an ambient temperature of 27°C, with only little within male variability. Because of the high repeatability of the “free-running” chirp period between song bouts of a male (0.69, Hartbauer et al., 2006) it is possible to classify males into slow, medium and fast signaling males using the population average as a reference. Behavioral experiments revealed that the likelihood of a male attaining the leader role in a song interaction with another male strongly depends on the difference between the individual “free-running” signal periods of opponents (Hartbauer et al., 2005). Only in the case of similar “free-running” chirp periods did leader and follower roles frequently change between two interacting males, whereas a difference of more than 150 ms was sufficient to increase the lead probability of the intrinsically faster singing male significantly. A similar correlation between the “free-running” chirp period and lead probability was also found in *Neoconocephalus nebrascensis* (Meixner and Shaw, 1986), *Necoconocephalus spiza* (Greenfield and Roizen, 1993) and the firefly *Pteroptyx cribellata* (Buck et al., 1981), which suggests this correlation to be a more general one. Additional entrainment experiments revealed that individual males with a shorter than average free-running signal period (<2 s) more frequently timed their chirps in advance to a conspecific pacer with a signal period of either 1.6 or 1.8 s.

## The neuronal basis for a preference of leader signals: evidence for a sensory bias?

Imagine a situation in the field, where synchronizing males of *M. elongata* are separated in space, so that leader and follower signals will arrive at a listening female from opposite directions. How are these two signals represented in the afferent auditory system of the female? A characteristic feature of the insect (and vertebrate) auditory pathway are direction-sensitive interneurons which receive excitatory synaptic inputs from the ipsilateral side but strong contralateral inhibition, such as those shown in Figure 2 (reviewed in Pollack, 1998). Bilateral pairs of interneurons may also make reciprocally inhibitory connections so that activity in one cell inhibits the corresponding cell on the other side and vice versa (Selverston et al., 1985). This has been proposed as the neural correlate for the lateralization behavior of these insects (Römer and Rheinlaender, 1989; Horseman and Huber, 1994a,b). Römer et al. (2002) tested the hypothesis that mutually inhibitory connections between an identified pair of auditory interneurons create strong asymmetries in the CNS in favor of the leading signal. The rationale behind the hypothesis was that the stimulus leading in time would activate one side of the auditory pathway and initiate strong contralateral inhibition on the opposite side, so that the response induced by the follower signal should be strongly reduced due to integration with contralateral inhibition. The effect would be a strong asymmetrical activation of neurons on both sides although the two stimuli only differ in their temporal relationship.

The results show clearly that time delays between 70 and 120 ms separating the signals of leader and follower are most effective in creating such asymmetries in the responses of this pair of interneurons. A further study with another auditory interneuron with T-shaped morphology (TN1 neuron) that also receives strong contralateral inhibition revealed even stronger asymmetries with leader—follower stimulation (Siegert et al., 2011). In this pair of interneurons, the response to follower signals was almost completely suppressed during presentation of leader signals. Time—intensity—trading experiments, in which the intensity of follower signals were traded against signal timing revealed that follower signals had to be more than 10 or 20 dB louder in order to compensate for a temporal advantage of signals leading by 70 or 140 ms, respectively. This neural advantage correlates with the behavioral observation that the choice of female *M. elongata* between two synchronizing males depends on how often, and by what time difference, one of the two males leads (Fertschai et al., 2007).

## Leader preference as a by-product of directional hearing?

The lateral inhibition of auditory neurons in grasshoppers, crickets, and katydids enhances the peripheral directionality of the ears by providing a contrast in the neuronal representation between both sides (review in Pollack, 1998; Hennig et al., 2004). It is thus highly likely that it evolved in the context of directional hearing. The same lateral inhibition may thus represent a case of a pre-existing sensory bias (reviewed Ryan, 1990; Ryan and Keddy-Hector, 1992) that may affect sexual selection through the female preference for leading signals. The core of pre-existing sensory bias models is that the evolution of male signals can be explained by properties of the sensory system of females predisposing them to “prefer” certain male signals in a choice situation.

One prerequisite of the sensory bias model, namely that the bias in the female nervous system must already exist before the male signal evolves, may hold for the auditory system of insects, and hearing animals in general, because lateral inhibition is one of the most common neural mechanisms in organisms equipped with two ears (Grothe, 2003; Schnupp and Car, 2009). However, with the evolution of such a neural mechanism, the leader male in an imperfect synchronous acoustic interaction in Mecopoda can “exploit” the mechanism of contralateral inhibition, in favor of the representation of his signal in listening females (the sensory exploitation hypothesis; Ryan and Rand, 1990, 1993; Ryan et al., 1990; Ryan, 1999).

However, although the ‘sensory bias hypothesis’ for the preference of leader signals in Mecopoda is an appealing one, two lines of evidence exist against it. One was provided in a recent phylogenetic study conducted in the genus *Neconocephalus* where, except in one species, discontinuously calling species synchronize their calls (Greenfield, 1990; Greenfield and Schul, 2008; Deily and Schul, 2009; Schul, unpublished observations), but females in this genus did not always show a strong leader preference in choice experiments (Greenfield and Schul, 2008). Furthermore, the sensory bias hypothesis would get some support if one could show that in distantly related insect species, where synchrony does not occur, the responses to follower signals in directionally sensitive interneurons are also suppressed. Our attempts in a locust (*Schistocerca gregaria*) and a cricket (*Gryllus bimaculatus*) were ambiguous, with a strong suppression in AN1 in the locust, but only weak to absent effect in AN1 of the cricket (Römer and Hirtenlehner, unpublished). These results argue against a pre-existing sensory bias affecting the temporal processing of temporally delayed signals. Therefore, it is likely that the evolution of chorus synchrony in *M. elongata* is influenced by other factors as well. A possible candidate for driving evolution toward chorus synchrony may constitute a parasitoid fly that homes in on *M. elongata* males by exploiting the calling song of males for host localization. In a field study where singing males have been collected some of them died as a result of infestation by tachinid flies; several pupae emerged out of the dead animals which subsequently developed into adult flies (Hartbauer and Siegert, unpublished). Lee et al. (2009) had shown before that the tachinid fly species *Ormia ochracea* exhibits a preference for the leader of identical chirps presented from different directions. If a similar preference exists in the parasitoid fly infesting Mecopoda, this preference may stabilize the follower role by exposing the leader of a chorus to a higher parasitation rate, producing a trade-off between sexual selection through mate choice, and natural selection via reduced survival of males singing as leaders in synchronous acoustic interactions.

## Processing of signals lasting for many seconds or minutes

In the past, the problem of time computation in the auditory system has almost exclusively been studied in the time domain from microseconds to hundreds of milliseconds, or a few seconds at the best. However, the duration of insect communication signals varies from less than a millisecond to many minutes (Reinhold, 2009). In some cases, such signals consist of repetitions of single syllables and short inter-syllable intervals for many seconds or minutes, in others of repetitions of song elements in “loop mode,” again for minutes to hours. The neuronal processing of such long lasting signals is virtually unexplored. From a functional point of view signals in the range of many minutes may be of particular interest, because the efficiency of sound production is generally rather low: only some part of the muscle energy used for calling is converted into acoustic energy contained in the call. Empirical data for insects report the efficiency of sound production in the range of 1.0% or even lower (Kavanagh, 1987; Prestwich, 1994). This makes long lasting acoustic signals attractive for sexual selection studies, because it has been widely accepted that sexual traits that increase individual fitness must have some costs balancing their benefit. Of course, a long duration signal, or one with many repetitive elements not just indicates the ability of a sender to invest much energy in signal production, but also causes signal redundancy, which may be particularly important in situations where receiver errors are likely, or when the transmission channel poses problems for reception due to background noise.

However, there may be problems associated with the proximate mechanism of evaluating such signals in the acoustic domain. Imagine, for example, the choice situation for a female of the *Mecopoda* complex, where males produce song bouts lasting for up to 20 min, each bout consisting of an amplitude-modulated part and a trill. The bouts of individual males do overlap non-randomly over the whole active period at night, and they also interfere with background noise of the nocturnal rainforest (Krobath et al., unpublished). Here, the mate sampling behavior would be important, i.e., how individuals gather information about potential mates and make decisions based on that information (Gibson and Langen, 1996; Jennions and Petrie, 1997). In particular in a sequential mate choice situation, it would require a memory system to evaluate differences in this time dimension properly. This is part of the general problem with processing acoustic signals, which “fade away” as soon as they are produced, in contrast to visual traits, which can be evaluated by the sensory system of receivers for long periods of time. Moreover, insects often communicate in choruses with many conspecific and heterospecific signalers, where it should be even more complicated to attribute these signals to particular sound sources in space.

Another problem is the assessment of a dynamic temporal character such as call rate, where females often prefer higher over lower rates. However, in a simultaneous choice situation the correct information about the call rate value accumulates slowly over time. If two males call at chirp rates of 120 and 140 chirps per min, a difference of one chirp can be perceived only after a time interval of about 3 s (Trobe et al., 2011). However, the probability of making a correct decision about the difference increases over time as information accumulates, and the slower the decision the more accurate it will be. This will create a dilemma for the female known as the speed–accuracy trade-off (Chittka et al., 2009). It is obvious that the issue of processing long lasting sound signals in insects requires more attention in the future, both at the behavioral and neurophysiological level.

### Conflict of interest statement

The authors declare that the research was conducted in the absence of any commercial or financial relationships that could be construed as a potential conflict of interest.
